# Supplementary material for: Mapping the evolution of fertility support policies in China: A content and instrumental analysis
Source: PLoS One. 2025 Oct 9;20(10):e0332137. doi: 10.1371/journal.pone.0332137 (PMC12510515; doi:10.1371/journal.pone.0332137)
Supplement: S1 Appendix — (ZIP) [file pone.0332137.s001.zip › S1 Appendix. 226 original policy documents/215-第十二届全国人民代表大会第二次会议关于2013年国民经济和社会发展计划执行情况与2014年国民经济和社会发展计划的决议(FBM-CLI-1-220743).doc]

第十二届全国人民代表大会第二次会议关于2013年国民经济和社会发展计划执行情况与2014年国民经济和社会发展计划的决议

**发布部门：** [全国人民代表大会](https://www.pkulaw.com/law/chl?Aggs.IssueDepartment=101)

**发布日期：**2014.03.13

**实施日期：**2014.03.13

**时效性：** [现行有效](https://www.pkulaw.com/law/chl?Aggs.TimelinessDic=01)

**效力级别：** [工作文件](https://www.pkulaw.com/law/chl?Aggs.EffectivenessDic=XA0105)

**法规类别：** [人大议事](https://www.pkulaw.com/law/chl?Aggs.Category=00106) [国家计划](https://www.pkulaw.com/law/chl?Aggs.Category=02404) [营商环境优化](https://dobusiness.pkulaw.com/)

第十二届全国人民代表大会第二次会议关于2013年国民经济和社会发展计划执行情况与2014年国民经济和社会发展计划的决议
（2014年3月13日第十二届全国人民代表大会第二次会议通过）

　　第十二届全国人民代表大会第二次会议审查了国务院提出的《关于2013年国民经济和社会发展计划执行情况与2014年国民经济和社会发展计划草案的报告》及2014年国民经济和社会发展计划草案，同意全国人民代表大会财政经济委员会的审查结果报告。会议决定，批准《关于2013年国民经济和社会发展计划执行情况与2014年国民经济和社会发展计划草案的报告》，批准2014年国民经济和社会发展计划。

关于2013年国民经济和社会发展计划执行情况与2014年国民经济和社会发展计划草案的报告
（--2014年3月5日在第十二届全国人民代表大会第二次会议上 国家发展和改革委员会）

各位代表：
　　受国务院委托，现将2013年国民经济和社会发展计划执行情况与2014年国民经济和社会发展计划草案提请十二届全国人大二次会议审议，并请全国政协各位委员提出意见。
　　**一、**2013年国民经济和社会发展计划执行情况
　　2013年，面对错综复杂的国内外形势，各地区、各部门按照党中央、国务院的决策部署，坚持稳中求进工作总基调，坚持统筹稳增长、调结构、促改革，坚持宏观政策要稳、微观政策要活、社会政策要托底的有机统一，依据十二届全国人大一次会议审议批准的国民经济和社会发展计划，扎实做好各方面工作，经济社会发展实现了良好开局，计划执行情况总体是好的。
　　(一)经济运行处于合理区间。保持政策定力，创新调控思路和调控方式，经济社会发展稳中有进、稳中向好。
　　一是经济运行总体平稳。初步核算，国内生产总值56．88万亿元，增长7．7％，完成预期目标。全国公共财政收入12．91万亿元，增长 10．1％，财政赤字1．2万亿元。全年新增人民币贷款8．89万亿元，年末广义货币供应量(M_2_)增长13．6％。规模以上工业企业实现利润6．28万亿元，比上年增长12．2％。重点领域、重要时段煤电油气运需求得到较好保障。
　　二是内需继续成为增长主动力。社会消费品零售总额23．78万亿元，增长13．1％，一些新的消费模式和消费领域表现出巨大活力，电子商务市场交易额超过10万亿元，增长25％以上。全社会固定资产投资44．71万亿元，增长 19．3％。民间投资增长23．1％，占固定资产投资(不含农户)比重达到63％，比上年提高1．8个百分点。内需对经济增长的贡献率达到 104．4％。
　　三是物价总水平基本稳定。全年居民消费价格上涨2．6％，控制在预期目标范围内。重要商品的收储、投放和进出口调节工作有序开展。生猪、蔬菜市场价格政策性保险试点积极推进。价格收费检查和反价格垄断执法力度加大，全年共查处价格违法案件3．44万起，实施经济制裁 31．25亿元。
　　(二)结构调整取得新进展。突出重点，有扶有控，着力提质增效升级，为经济发展增添后劲。
　　一是产业结构调整稳步推进。技术创新和培育新兴产业成效显著。研究与试验发展经费支出占国内生产总值比例达到2．09％，完成预期目标。嫦娥三号探测器、神舟十号载人飞船、蛟龙号载人深潜器等重大创新成果举世瞩目。节能环保、新一代信息技术、新能源、高端装备制造、新材料等战略性新兴产业发展加快，第四代移动通信开始大规模商用。高技术制造业增加值增长 11．8％，比规模以上工业增加值增速高2．1个百分点。传统产业改造升级加快，化解产能过剩矛盾力度加大。下发了[坚决遏制产能严重过剩行业盲目扩张的通知](https://www.pkulaw.com/chl/c0b669f0704e8361bdfb.html)，出台了[化解产能严重过剩矛盾的指导意见](https://www.pkulaw.com/chl/0e329cc15ba3af23bdfb.html)和配套政策措施，着力化解钢铁、水泥、电解铝、平板玻璃、船舶等行业产能严重过剩矛盾。服务业发展环境优化。出台了促进信息消费、养老服务业、健康服务业发展等政策意见和全国物流园区发展规划，继续推进服务业综合改革试点，服务业增加值增长8．3％，占国内生产总值比重达到46．1％，首次超过第二产业。基础产业加快发展。新建铁路投产里程5586公里，其中高速铁路1672公里；新建公路里程 7．03万公里，其中高速公路8260公里；建成万吨级以上泊位110个；新增民航通航机场10个。能源生产保持稳定，原煤、原油、天然气产量分别增长0．8％、1．8％和9．4％，能源结构进一步优化，非化石能源占一次能源消费比重9．8％，比上年提高0．4个百分点。
　　二是城乡发展一体化有序推进。认真落实强农惠农富农政策，中央预算内投资用于“三农”建设的比重超过一半。农业综合生产能力增强，粮食总产量达到6．02亿吨，实现“十连增”，肉蛋奶、果菜鱼等农产品供应稳定。农业科技进步贡献率达到55．2％，比上年提高0．7个百分点。农村安全饮水普及率提高到88．5％，新建和改造农村电网线路21．1万公里，新建改建农村公路21万公里，新增农村沼气用户80万户，改造农村危房266万户。支持建设粮食收储仓容440万吨、农产品批发市场114个。中央对积极稳妥推进新型城镇化作出全面部署，国家新型城镇化规划编制基本完成。常住人口城镇化率为 53．73％，比上年提高1．16个百分点。
　　三是区域发展协调性增强。西部地区交通、水利、能源等重大基础设施建设得到加强，新开工重点工程20项，投资总规模3265亿元，内陆沿边地区开发开放步伐加快。东北地区等老工业基地振兴战略深入推进，城区老工业区搬迁改造试点、独立工矿区改造搬迁工程试点启动实施，全国老工业基地调整改造规划、资源型城市可持续发展规划出台。中部地区长江中游城市群、中原经济区发展的集聚效应进一步显现，承接产业转移示范区建设进展顺利。东部地区产业转型升级加快，海洋经济发展试点有序推进。主体功能区规划深入实施。区域合作和对口支援顺利推进，对革命老区、民族地区、边疆地区和贫困地区的支持继续加大，集中连片特殊困难地区区域发展与扶贫攻坚规划顺利实施。玉树灾后恢复重建任务全面完成，四川芦山和甘肃岷县漳县地震灾后恢复重建进展顺利。
　　四是节能减排和环境保护步伐加快。国家适应气候变化战略、循环经济发展战略及近期行动计划、[加快发展节能环保产业的意见](https://www.pkulaw.com/chl/627cb162bd1d0e91bdfb.html)发布实施，大气污染防治行动计划颁布施行。重点领域和重点企业节能减排积极推进，重点流域和区域水环境综合治理扎实开展。天然林资源保护、京津风沙源治理、石漠化治理、退耕还林、退牧还草等生态建设深入推进，完成造林面积609万公顷。资源节约利用和环境保护各项指标基本实现：单位国内生产总值能耗和二氧化碳排放量分别下降 3．7％和4．36％，二氧化硫、化学需氧量、氨氮、氮氧化物排放量分别下降3．48％、2．93％、 3．14％、4．72％，万元工业增加值用水量下降 5．7％，城市污水处理率和城市生活垃圾无害化处理率分别达到87．9％和85．8％。积极建设性参与应对气候变化国际谈判和交流合作。
　　(三)改革开放力度加大。经济体制改革全面推进，为稳增长和调结构创造良好体制机制环境。
　　一是行政体制改革成效明显。国务院机构改革顺利进行，行政审批制度改革深入开展，取消和下放416项行政审批等事项，出台了严格控制新设行政许可的措施。[政府向社会力量购买服务的指导意见](https://www.pkulaw.com/chl/7d14fc5cb7bf4a89bdfb.html)发布实施。公司注册资本登记制度改革加快推进。不动产统一登记制度开始建立。
　　二是财税金融改革深入推进。交通运输业和部分现代服务业营改增试点在全国推开，全年减轻企业税负超过1400亿元。提高小微企业增值税和营业税起征点，600多万户企业受益。贷款利率管制全面放开。中小企业股份转让系统试点扩大至全国。信贷资产证券化试点扩大。新股发行体制改革、加强中小投资者权益保护等指导意见出台。创新了企业债券品种和审批方式。
　　三是投资体制改革取得积极进展。重新修订政府核准的投资项目目录，需报中央管理层面核准的企业投资项目减少60％。改进中央预算内投资补助贴息管理办法，下放了31类点多面广量大单项资金少的中央预算内投资补助贴息项目安排权。铁路投融资体制改革深入推进。对民间投资36条及42项实施细则落实情况进行了第三方评估，鼓励民间投资健康发展的政策措施进一步落实和完善。
　　四是资源性产品价格形成机制不断完善。推出成品油价格形成新机制，出台了支持油品质量升级的价格政策，调整了非居民用天然气门站价格，提高了可再生能源电价附加和环保电价标准，完善核电、水电和光伏发电上网电价机制，顺利实现电煤价格并轨。
　　五是农村改革积极推进。全国农村集体土地所有权确权登记颁证工作基本完成，农村土地承包经营权确权登记试点范围扩大到105个县 (市、区)。国有林场改革试点在7个省展开。
　　六是社会领域改革稳步开展。深化收入分配制度改革的若干意见发布实施。28个省(区、市)启动实施城乡居民大病保险试点，县级公立医院综合改革试点扩大到1000多家。前两批非时政类报刊出版单位转企改制试点基本完成。食品药品监管体制进一步完善。
　　与此同时，国有企业和重点行业改革继续深化，黑龙江省“两大平原”现代农业综合配套改革试验启动实施，对11个国家综合配套改革试验区进行了第三方评估和自评估。
　　对外开放继续深化。外贸进出口总额增长 7．6％，比上年加快1．4个百分点。全年非金融类外商直接投资1176亿美元，增长5．3％，服务业实际使用外资比重首次过半，外商投资加快向中西部地区梯度转移。非金融类境外直接投资 902亿美元，增长16．8％。中国上海自由贸易试验区挂牌运行。推动世贸组织多哈回合谈判达成“早期收获”协议，与冰岛、瑞士签署自贸协定。积极落实丝绸之路经济带、21世纪海上丝绸之路战略构想，协调推进孟中印缅经济走廊、中巴经济走廊建设。
　　(四)人民生活继续改善。加大投入力度，完善政策措施，促进经济社会协调发展。
　　一是就业保持总体稳定。城镇新增就业 1310万人，年末城镇登记失业率4．05％，超额完成预期目标。高校毕业生就业创业扶持政策得到较好落实，面向就业困难人员的就业援助制度进一步健全，公共就业服务设施建设继续推进。
　　二是居民收入继续增长。城镇居民人均可支配收入和农村居民人均纯收入分别实际增长7％和9．3％。农村居民收入增速连续4年快于城镇居民，农村贫困人口减少1650万人，城乡居民收入差距继续缩小。
　　三是社会保障制度覆盖面不断扩大。基本养老保险和医疗保险制度的城乡统筹加快推进，城乡低保制度进一步规范，临时救助制度在26个省份建立。社会救助和保障标准与物价上涨挂钩的联动机制得到完善。年末参加城镇基本养老和新型农村社会养老保险人数分别达到3．46亿人和4．74亿人，增加2083万人和1082万人。企业退休人员基本养老金水平提高10％，城镇居民医保和新农合参保财政补助标准由每人每年 240元提高到280元。
　　四是教育事业稳步发展。农村学前教育推进工程继续实施，义务教育学校标准化建设深入推进，贫困地区义务教育薄弱学校基本办学条件持续改善。进城务工人员随迁子女接受义务教育后在当地参加升学考试政策得到较好落实。农村学生上重点高校比例提升。九年义务教育巩固率 92．3％，提高0．5个百分点；高中阶段教育毛入学率86％，提高1个百分点；普通高等学校招生699．8万人，研究生招生77．9万人，其中全日制研究生招生61．1万人。
　　五是医疗卫生服务体系继续完善。公共卫生服务体系、医疗服务体系、计划生育服务体系、全科医生培养基地和医药卫生信息化建设得到加强。人均基本公共卫生服务经费标准从25元提高到30元。每千人口医疗卫生机构床位数4．55张，增长7．3％。人口自然增长率4．92‰，实现计划目标。
　　六是公共文化服务体系不断健全。广播电视村村通、文化信息资源共享等文化惠民工程扎实推进，国家文化和自然遗产保护设施建设专项继续实施。[旅游法](https://www.pkulaw.com/chl/702117f6e16054f3bdfb.html)和国民旅游休闲纲要出台，全年旅游总收入2．95万亿元，增长14％。群众体育保障水平提高，全年新增体育场地5．18万个。
　　七是保障性安居工程建设加快。全年基本建成城镇保障性安居工程住房544万套，新开工 666万套，均超过计划目标。
　　在国内外环境错综复杂的情况下，经济保持平稳发展，社会大局和谐稳定，成绩来之不易。这是党中央、国务院科学决策、正确领导的结果，是各地区、各部门齐心协力、狠抓落实的结果，是全国各族人民艰苦奋斗、共同努力的结果。
　　在看到成绩的同时，我们也清醒地认识到，经济社会发展还面临不少矛盾和困难。世界经济继续呈缓慢复苏态势，仍存在不稳定不确定因素。国内发展中不平衡不协调不可持续的问题依然突出，稳中向好的基础尚不稳固，一是经济增长的动力不足。新的消费增长点不多，投资接续能力偏弱，出口形势仍然严峻。二是结构优化升级任务依然艰巨。部分行业产能过剩问题严重，科研成果向生产力转化不足，一些企业生产经营困难。三是制约农业稳定发展的问题凸显。资源环境约束趋紧，农田水利基础设施仍然薄弱，现有生产经营体制不完全适应现代农业发展要求，农业生产成本上升、比较效益下降。四是资源消耗和环境污染问题突出。节能减排形势严峻，特别是大范围多频次的雾霾天气，严重影响人民群众的生活和健康。五是财政金融潜在风险不容忽视。部分地方和行业债务负担较重，一些企业负债率过高，金融机构不良资产有所增加，流动性风险管理难度增大。同时，结构性就业矛盾突出，部分大城市住房价格上涨过快，房地产领域存在风险隐患，食品药品质量、安全生产、收入分配、征地拆迁等方面也存在一些比较突出的问题。对此，我们一定高度重视，切实采取措施，认真加以解决。

　　**二、**2014年经济社会发展的总体要求、主要目标和政策取向
　　2014年是全面深化改革的第一年，也是完成“十二五”规划目标任务的关键一年。要全面贯彻落实党的十八大和十八届二中、三中全会精神，按照党中央、国务院决策部署，坚持稳中求进工作总基调，把改革创新贯穿于经济社会发展各个领域各个环节，保持宏观经济政策连续性和稳定性，着力激发市场活力，加快转方式调结构，加强基本公共服务体系建设，着力保障和改善民生，切实提高经济发展质量和效益，促进经济持续健康发展、社会和谐稳定。
　　按照上述总体要求，综合考虑需要和可能，提出2014年经济社会发展的主要预期目标：
　　--保持经济合理增长。国内生产总值预期增长7．5％左右。这是统筹考虑抓改革、促调整、惠民生、防风险等各方面因素确定的。一方面，发展仍是解决我国所有问题的关键，推进经济结构调整、稳定和扩大就业、保障和改善民生都需要合理的速度，进一步减少贫困、实现全面建成小康社会目标，更要靠发展。另一方面，今年稳增长的有利条件不少，全面深化改革将进一步激发经济发展动力和活力，稳增长政策措施的效应会持续显现，消费、投资、外需均有一定支撑。同时，我国已经到了加快转变经济发展方式的关键阶段，转型升级的任务很重，速度也不宜定得过高。7．5％左右是一个经济增长的预期目标，是弹性的、引导性的，各地应根据自身实际，合理确定增长速度，决不能摘层层加码和盲目攀比。
　　--注重提质增效升级。现代服务业加快发展，服务业增加值占国内生产总值的比重继续提升。研发投入占国内生产总值的比例提高，传统产业加快改造升级，战略性新兴产业健康发展。
　　新型城镇化有序推进，区域发展更趋协调。生态文明建设取得积极进展，单位国内生产总值能耗下降3．9％以上，单位国内生产总值二氧化碳排放量下降4％，二氧化硫、化学需氧量、氨氮排放量均下降2％，氮氧化物排放量下降5％，万元工业增加值用水量下降5．2％。加快经济转型升级，是我们面临的艰巨而紧迫的任务，决不能以牺牲结构和资源环境为代价换速度。提出上述组合目标，就是为了引导各方面把精力集中到优化产业结构、推进新型城镇化、促进区域协调发展、加强生态文明建设上来，着力解决重大结构性问题，提高经济发展的质量和效益。
　　--保持物价基本稳定。居民消费价格涨幅控制在3．5％左右。这一目标既考虑了各方面涨价因素和人民群众的承受力，也为推进改革和调整结构留出了一定空间。一方面，当前我国总供给和总需求基本平衡，粮食连年增产，绝大多数产品供应充足，保持物价总水平基本稳定具备较好基础和诸多有利条件。另一方面，今年物价仍面临一定上涨压力，既有上年的翘尾影响，也有新涨价因素，同时还要推进资源性产品价格改革，实现这一目标需要付出不小努力。
　　--突出民生优先。城镇新增就业1000万人以上，城镇登记失业率控制在4．6％以内，大城市调查失业率稳定在合理水平。城乡居民收入增长与经济增长基本同步，社会保障体系进一步健全。基本公共服务均等化继续推进。人口自然增长率控制在6．5‰以内。城镇保障性安居工程住房基本建成480万套、新开工700万套以上。今年城镇新增就业预期目标比去年增加100万人，主要是为了更好地满足城镇新成长劳动力就业需要，适应今年高校毕业生继续增加、就业压力较大的现实。同时，随着国内生产总值基数增大和服务业发展加快，每增长一个百分点带动的就业数量增多，实现这样的目标，也是有条件的。
　　--保持国际收支基本平衡。外贸进出口总额增长7．5％左右，服务贸易发展加快。对外投资持续扩大，利用外资总量保持稳定，结构进一步优化。促进进出口稳定增长政策效应的继续发挥，有利于增强企业信心，保持外贸稳定增长。但同时，国际市场竞争加剧，投资和贸易保护主义明显抬头，出口形势仍然十分严峻，巩固和扩大外需面临不小压力。
　　实现上述经济社会发展预期目标，做好 2014年经济工作，核心是坚持稳中求进、改革创新。为此，要继续实施积极的财政政策和稳健的货币政策，不断完善调控方式和手段，增强调控的前瞻性、针对性、协同性。
　　继续实施积极的财政政策。今年拟安排全国财政赤字1．35万亿元，比上年预算增加1500亿元，其中中央财政赤字9500亿元，由中央代地方发债4000亿元，赤字率稳定在2．1％。政策重点是：第一，完善结构性减税政策，扩大营改增试点行业范围，扩展小微企业税收优惠政策，实施鼓励企业年金和职业年金发展的财税政策。第二，调整优化支出结构，重点用于保障和改善民生，向农业、教育、社保、卫生、环保、科技等领域倾斜。从严控制“三公”经费支出，努力降低行政运行成本。第三，用好新增资金，盘活存量资金，建立结转结余资金定期清理机制，加大资金统筹使用力度。第四，加强地方政府性债务管理，加快建立以政府债券为主体的地方政府举债融资机制，推动形成规范合理的中央和地方政府债务管理及风险预警机制。
　　继续实施稳健的货币政策。今年广义货币供应量(M_2_)预期增长13％左右。政策重点是：第一，综合运用多种货币政策工具，加强宏观审慎管理，引导货币信贷和社会融资规模平稳适度增长，保持适度流动性，创造稳定的货币金融环境。第二，优化金融资源配置，用好增量，盘活存量，加快资金周转。鼓励和引导金融机构加大对“三农”、小微企业、结构调整的金融支持，提高金融服务实体经济和支持产业转型升级的能力。促进金融市场、金融产品、市场主体多元化发展，提高直接融资比重。第三，完善金融监管协调机制，加强对信贷、债券、理财、同业和信托等业务的风险监管，守住不发生系统性和区域性金融风险的底线。密切监测跨境资本流动，防止大规模资本异常流动对经济和金融运行造成大的冲击。保持人民币汇率在合理均衡水平上的基本稳定。

　　**三、**2014年经济社会发展的主要任务
　　今年要着力抓好9方面工作。
　　(一)全面深化改革扩大开放。(1)行政体制改革。进一步取消和下放行政审批事项，建立行政审批事项目录清单制度并向社会公开。加快建立纵横联动协同管理机制，改进政府监管方式。继续推进工商登记制度由先证后照改为先照后证。推进公务用车制度改革。开展行业协会商会与行政机关脱钩试点。(2)国有企业改革。准确界定不同国有企业功能，推进自然垄断行业改革。鼓励非公有制企业参与国有企业改革，积极发展混合所有制经济。完善国有资产管理体制。 (3)财税体制改革。推进政府和部门预算决算公开，提高一般性转移支付比例，减少专项转移支付项目。完善国有资本经营预算，提高中央企业国有资本收益上缴公共财政比例。调整消费税征收范围、环节和税率，推动煤炭资源税改革。研究逐步理顺中央和地方事权。(4)金融体制改革。建立存款保险制度，稳步推进政策性金融机构改革、民间资本发起设立中小型银行等金融机构，健全金融机构风险处置机制。推动股票发行制度改革。推进利率市场化。促进互联网金融健康发展。完善汇率市场化形成机制，扩大人民币跨境使用，推进资本项目可兑换。(5)资源性产品等价格改革。适时调整风电上网电价，开展输配电价改革试点，完善煤电价格联动机制和抽水蓄能电价机制。适时调整非居民用存量天然气门站价格。建立健全居民用水用气阶梯价格制度。提高主要污染物排污费标准和部分水利工程供水价格。完善铁路运价机制。(6)投资体制改革。加快出台政府投资条例。修订企业投资、外商投资、境外投资核准管理办法。改进审批方式，简化审批内容，优化审批流程，提高服务效率和透明度。实施铁路投融资体制改革。(7)农村体制改革。慎重稳妥进行农村土地制度改革试点，抓紧土地承包经营权及农村集体建设用地使用权确权登记颁证工作。有序推进承包地经营权流转，发展多种形式适度规模经营。完善征地制度，切实保障农民公平分享土地增值收益。健全农业社会化服务体系，扩大农业生产全程社会化服务试点范围。完善集体林权制度改革，推进国有林场和国有林区改革。继续开展国有农场办社会职能改革。(8)社会事业改革创新。推进职业教育、考试招生等改革，鼓励社会力量兴办教育。扩大公立医院综合改革试点，巩固完善基本药物制度和基层医疗卫生机构运行新机制。推动文化企业跨地区、跨行业、跨所有制兼并重组，允许社会资本以控股形式参与国有影视制作机构、文艺院团改制经营。推进养老服务业综合改革试点。结合社会事业改革，放开一批服务价格和收费。同时，扎实推进国家综合配套改革试点。
　　深入实施互利共赢开放战略，提升开放层次和水平。(1)优化进出口结构。巩固传统出口优势，发挥大型成套装备对关联行业和服务出口的带动作用，加大信用保险、贸易融资等支持力度。引导加工贸易转型升级。妥善应对贸易摩擦。增加国内短缺的技术、资源、产品进口。积极发展服务贸易。(2)放宽外商投资市场准入。深化外商投资管理体制改革，探索对外商投资实行准入前国民待遇加负面清单的管理模式。加快金融、教育、文化、医疗等服务业领域有序开放。推进中国上海自由贸易试验区先行先试。完善外资安全审查制度。2014年非金融类外商直接投资预期达到1217亿美元，增长3．5％。(3)支持符合条件的企业加快走出去。完善境外投资支持政策，改革和简化外汇管理，有效利用外汇储备支持境外投资，深化境外能源资源、农业开发合作与投资，引导优势产业走出去。2014年非金融类境外直接投资预期达到992亿美元，增长10％。(4)强化多边双边及区域经济合作。扩展内陆沿边开放，统筹推进内陆地区国际大通道建设。积极推进丝绸之路经济带、21世纪海上丝绸之路建设，推动孟中印缅经济走廊、中巴经济走廊建设，加强与周边国家基础设施互联互通。继续推进中韩、中澳等双边重点自贸区谈判和区域全面经济伙伴关系。
　　(二)着力扩大内需。充分发掘消费潜力。 2014年社会消费品零售总额预期增长14．5％。 (1)增强居民消费能力。合理调整国民收入分配格局，抓紧出台深化收入分配制度改革实施细则，努力增加城乡居民特别是低收人群体收入。 (2)大力培育和发展新的消费热点。实施“宽带中国”战略，支持第四代移动通信网络建设和业务发展，推广“三网融合”，开展电子商务示范城市建设，扩大物联网应用，促进信息消费。继续实施节能产品惠民工程，推动节能环保和再生产品消费。支持社会力量举办养老和健康服务机构，积极发展养老、健康等服务消费。出台推进文化创意和设计服务与相关产业融合发展以及促进旅游消费的指导意见，进一步落实带薪休假制度，推动文化、旅游、体育等消费健康发展。 (3)优化消费环境。完善城乡流通网络，加快发展城市共同配送和农村配送，促进快递服务与网络零售协同发展，建立健全最严格的食品药品监管制度，推进食品安全放心工程，突出抓好婴幼儿配方乳粉、肉制品等重点行业综合整治。大力整顿和规范市场秩序，依法严厉打击制售假冒伪劣产品、价格欺诈等不法行为。建立健全社会征信体系，推进信用信息共建共享。
　　促进投资稳定增长和结构优化。2014年全社会固定资产投资预期增长17．5％，中央预算内投资拟安排4576亿元。(1)释放民间投资潜力。抓好民间投资36条及实施细则的落实工作。抓紧清理和修订有关民间投资的行政法规、部门规章及规范性文件，制定清晰透明、公平公正、操作性强的市场准入规则。尽快在金融、石油、电力、铁路、电信、资源开发、公用事业等领域，向民间资本推出一批符合产业导向、有利于转型升级的项目，发挥示范带动效应。(2)优化政府投资结构。抓紧逐步退出用于一般竞争性企业项目投资，适当减少可利用社会资金的经营性基础设施投资，压缩小型分散投资，停止建设楼堂馆所等投资，重点支持全局性、基础性、战略性的重大项目，集中力量办大事。中央预算内投资主要投向保障性住房及配套设施和棚户区改造，新增千亿斤粮食工程和农村民生，水利、中西部铁路等重大基础设施，节能减排和环境治理，重大核心关键技术开发应用等自主创新和转型升级工程，教育、医疗、社会养老、食品药品安全等社会事业和社会管理，少数民族、边疆地区发展等领域。(3)抓好重点项目。在“十二五”规划《纲要》实施中期评估的基础上，加快实施“十二五”规划明确的重点建设任务，扎实推进在建项目建设。积极推动项目前期工作，充实重大项目储备。
　　(三)促进农业稳定发展。(1)保持主要农产品生产稳定发展。加快实施全国新增千亿斤粮食生产能力规划，着力建设区域化、规模化、集中连片的商品粮生产基地。加快农业科技创新和推广，提高单产水平。加快发展现代种业和农业机械化。支持新疆优质棉生产基地建设。继续实施全国蔬菜产业发展规划，推进生猪、奶牛、肉牛、肉羊标准化规模养殖，加强动物疫病防控。支持渔政渔港建设和海洋渔船更新改造。推进农产品质量安全检验检测体系建设。2014年预期粮食总产量5．5亿吨以上。(2)完善和强化农业支持保护制度。中央预算内投资继续向“三农”倾斜。小麦、稻谷最低收购价平均每50公斤分别提高6元和3．7元。继续执行玉米、油菜籽、食糖临时收储制度。启动东北和内蒙古大豆、新疆棉花目标价格补贴试点。扩大农业补贴资金规模，新增补贴向粮食等重要农产品、新型农业经营主体、主产区倾斜。在有条件的地方，开展按实际粮食播种面积或产量对生产者补贴试点，提高补贴精准性、指向性。完善政策性农业保险制度。增加对商品粮大省和粮油猪生产大县的奖励补助，扶持牛羊肉生产，完善粮食主产区利益补偿机制。(3)加强农业农村基础设施建设。实施全国高标准农田建设总体规划，加强骨干水源、引调水工程、大型灌区等重点水利建设，抓好江河治理和病险水库水闸除险加固，推进防洪抗旱等防灾减灾体系建设。编制农业环境突出问题治理总体规划，加强耕地重金属污染、地下水严重超采区治理，加大农业面源污染防治力度。继续推进农村饮水安全工程、农村电网升级改造和县乡村公路建设，优化发展农村沼气，实施农村危房改造，加快农村电信基础设施建设。
　　(四)积极稳妥推进新型城镇化。2014年全国常住人口和户籍人口城镇化率预期分别达到 54．6％和37．1％。(1)有序推进农业转移人口市民化。统筹制定户籍、土地、资金、住房、基本公共服务等方面配套政策，促进有能力在城镇稳定就业和生活的常住人口有序实现市民化。研究破解城市内部二元结构的对策措施。推进城乡基本公共服务均等化。(2)优化城镇化布局和形态。国家编制实施重点城市群发展规划，各地因地制宜编制和实施本地区新型城镇化规划，促进大中小城市和小城镇协调发展。规范新城新区发展。(3)提高城市可持续发展能力。强化城市产业就业支撑，建立多元可持续的资金保障机制，加强市政公用设施和公共服务设施建设，提高对人口集聚和服务的支撑能力。(4)完善城镇化体制机制。围绕建立农业转移人口市民化成本分担、多元化城镇建设投融资等机制，在不同区域开展不同层级、不同类型的试点，加快形成有利于城镇化健康发展的制度环境。
　　(五)加强价格调控监管。(1)保障供给。合理把握粮、油、棉花、食糖、猪肉、化肥等收储投放力度、时机和节奏。实施好北方大城市冬春蔬菜储备制度。加快粮棉等大宗农产品仓储物流设施建设，积极发展农产品冷链物流，推进农产品批发市场升级改造。落实好“米袋子”省长负责制、“菜篮子”市长负责制。(2)完善价格调控机制。认真执行生猪等价格调控预案，继续开展生猪价格政策性保险试点。扩大基本蔬菜品种价格政策性保险试点，建立蔬菜价格稳定长效机制。巩固和完善价格调节基金制度。推动重要商品和服务价格指数体系建设。(3)加强价格监管。开展教育、医疗、银行等行业价格收费检查。清理取消违规收费项目。加大反价格垄断工作力度。(4)健全物价补贴联动机制。完善并及时启动社会救助和保障标准与物价上涨挂钩的联动机制，缓解物价上涨对困难群众生活的影响。
　　(六)促进产业结构优化升级。(1)推动重点产业布局调整优化。出台关于重点产业布局调整和产业转移的指导意见，优化沿海、沿边和内陆地区产业布局，推动城市高污染企业环保搬迁。促进产业集聚集约发展，推动工业园区和产业集群转型升级。(2)着力化解产能严重过剩矛盾。坚决遏制新增违规产能，多策并举优化存量产能。加快建立和完善以市场为主导的化解产能严重过剩矛盾长效机制。提高并严格执行能耗、环保和安全等行业准入标准，清理各种优惠政策，加快淘汰落后产能。对电解铝、水泥等行业实行阶梯电价政策。(3)深入实施创新驱动发展战略。2014年研究与试验发展经费支出占国内生产总值比例预期达到2．17％。加快科技体制改革。加大政府对基础研究、前沿技术、社会公益技术、重大共性关键技术的投入。加快国家科技重大专项的实施及成果应用。完善国家自主创新示范区先行先试政策，落实企业技术创新普惠政策，启动实施创新企业百强工程。深入推进重大科技基础设施和知识创新工程建设，在新一代信息技术、环保等重点产业建设一批国家工程中心和工程实验室。大力发展战略性新兴产业，出台支持云计算、民用空间基础设施发展的政策规划，组织实施重大产业创新发展工程和应用示范工程，推进北斗导航等自主技术产业化。深入实施新兴产业创投计划，整合设立国家新兴产业创业投资引导基金。加快发展集成电路、下一代互联网、移动互联网、物联网等产业，加强宽带和网络安全基础设施建设，推动智慧城市建设，全面实施信息惠民工程。(4)加强基础产业。推进铁路特别是中西部地区铁路建设，引导城市轨道交通有序发展，加快推进国家高速公路“断头路”、普通国道瓶颈路段和集中连片特困地区农村公路建设，加快长江等内河高等级航道建设，优化沿海港口布局，有序推进民航基础设施建设。合理控制能源消费总量，改善能源结构，鼓励发展风能、太阳能，开工一批水电、核电项目，加强天然气、煤层气、页岩气勘探开采与应用。(5)加快发展服务业。出台加快发展生产性服务业促进产业结构调整升级的指导意见。继续推进国家服务业综合改革试点和示范区建设。积极发展研发设计、现代物流、金融租赁、节能环保服务、检验检测等重点行业。出台物流业发展中长期规划，开展物流园区示范。2014年服务业增加值预期增长8．3％。
　　(七)构建各具特色、协调联动的区域发展格局。(1)继续深入实施区域发展总体战略。加快制定优先推进西部大开发的政策性文件，进一步加强基础设施建设，大力发展特色优势产业，启动实施新一轮退耕还林，扩大退牧还草范围。出台全面振兴东北地区等老工业基地若干意见，统筹推进全国老工业基地、城区老工业区更新改造和资源型城市、独立工矿区转型发展。全面落实中部崛起战略，深入推进“三基地、一枢纽”建设，加快中西部地区承接产业转移示范区和产业园区建设。推进东部地区率先发展，支持前海、南沙、横琴、平潭等重大平台建设，继续推进海洋经济发展试点。(2)加快培育新的经济支撑带。依托长江建设中国经济新支撑带，推进长三角地区经济一体化，深化泛珠三角区域经济合作，支持长江中游城市群一体化发展，促进中原经济区加快发展，深化东北四省区合作。深入推进京津冀地区协同发展，加快环渤海地区转型升级。推动云南、广西等地向南亚、东南亚开放，支持西北地区向西开放。(3)增强欠发达地区发展能力。支持西藏、新疆和四省藏区等跨越式发展，落实扶持革命老区振兴发展的规划和政策，继续实施以工代赈和易地扶贫搬迁规划，支持集中连片特殊困难地区发展，加大对口支援力度。今年再减少农村贫困人口1000万人以上。(4)加快落实主体功能区制度。完善财政、投资、产业、土地、环境等配套政策和政绩考核评价体系，推动省级主体功能区规划发布实施，开展主体功能区建设试点示范。
　　(八)扎实推进生态文明建设。(1)强化制度保障。研究制定加快推进生态文明建设的意见和加强应对气候变化工作的政策性文件。开展生态文明先行示范。建立健全生态补偿机制。完善污染者付费和矿产资源开发补偿制度、生态环保责任追究制度、环境损害赔偿制度。建立生态红线管控制度、企业环境信用评价制度。(2)强力推进节能减排。强化节能减排目标责任制和问责制，实行能源消费总量和能耗强度“双控”考核。实施节能减排重点工程，加强工业、建筑、交通和公共机构等重点领域节能减排和监管，深入开展万家企业节能低碳行动，加快重点用能单位能耗在线监测系统建设。启动实施“能效领跑者”制度，在政府投资的公益性建筑和2万平方米以上公共建筑强制执行绿色建筑标准。落实[加快发展节能环保产业的意见](https://www.pkulaw.com/chl/627cb162bd1d0e91bdfb.html)。推进节能减排科技专项行动。持续开展节能减排全民行动。(3)大力发展循环经济。落实循环经济战略及近期行动计划。开展循环经济示范城市(县)建设，深化园区循环化改造，加快再生资源回收利用产业化。继续开展国家“城市矿产”示范基地、餐厨废弃物资源化利用和无害化处理试点，实施第二批资源综合利用“双百工程”，推进粮棉主产区秸秆综合利用。整顿矿业开发秩序，发展绿色矿业。开展海水淡化试点示范。(4)狠抓环境治理和生态建设。加大大气污染防治行动计划实施力度，以细颗粒物(PM_2.5_)和可吸入颗粒物 (PM_10_)治理为突破口，以京津冀、长三角、珠三角等地区为重点加强区域联防联控，完善鼓励脱硫、脱硝、除尘改造以及炼油企业升级改造的经济政策。继续推进重点流域和区域水环境综合整治，制定实施清洁水行动计划。加强土壤污染治理。加快城镇污水、垃圾处理设施等工程建设。开展森林草原资源保护和建设，推进京津风沙源、岩溶石漠化、黄土高原、青藏高原等重点区域综合治理。全面推行清洁生产。(5)做好应对气候变化各项工作。深入推进低碳省区和低碳城市、低碳工业园区、低碳社区等试点示范，研究建立全国碳排放权交易市场，建立温室气体统计核算体系，加强应对气候变化能力建设。落实国家适应气候变化战略。建设性参与气候变化国际谈判，继续开展应对气候变化国际交流与务实合作。
　　(九)切实做好保障和改善民生工作。(1)实施更加积极的就业政策。完善促进就业税收扶持政策，落实促进高校毕业生就业创业的各项政策，做好未就业毕业生的就业服务。加强对就业困难人员的帮扶，抓好化解产能过剩中出现的下岗再就业工作。强化农民工职业技能培训和公共就业服务。加强基层就业服务能力建设。(2)统筹推进城乡社会保障体系建设。建立统一的城乡居民基本养老保险制度，提高企业退休人员基本养老金水平。加快城乡居民基本医疗保险制度整合，提高医保待遇水平，推进城乡居民大病保险和异地就医即时结算。完善城乡低保制度，全面建立临时救助制度。加强社会保障公共服务能力建设。(3)加快发展社会事业。推进义务教育均衡发展，改善贫困地区义务教育薄弱学校基本办学条件，推进进城务工人员子女平等接受教育，继续提高重点高校招收农村学生比例。加快构建以就业为导向的现代职业教育体系。2014年，普通高等学校计划招生698万人，研究生招生 80．2万人，其中全日制研究生招生63．2万人。加强城乡医疗和公共卫生服务体系建设，健全突发公共卫生事件应急和重大疾病防控机制，支持社会资本办医疗机构。推进实施单独两孩政策，提升人口和计划生育服务能力。实施好中国妇女儿童发展纲要和国家贫困地区儿童发展规划。加强残疾人康复和托养设施建设。继续实施文化惠民工程，加快新闻出版、广播电视、文学艺术等公共文化事业和文化产业发展，加强文化自然遗产保护利用。繁荣和发展哲学社会科学。改善旅游基础设施条件，积极发展红色旅游。建立健全全民健身公共服务体系。(4)做好住房保障和房地产市场调控工作。坚持分类指导、分步实施、分级负责，加快解决中低收入群众基本住房问题。继续开展城镇保障性住房建设、棚户区改造和农村危房改造，加大配套基础设施建设的支持力度，推进廉租房和公租房并轨运行，加强分配和运营维护等后续管理。落实好房地产市场调控政策，推动建立房地产市场健康发展长效机制。 (5)创新社会治理和公共服务。整合基层公共服务资源，健全基层综合服务管理平台，推进政府公开透明向社会力量购买服务，坚决纠正损害群众利益的行为。
　　继续深化内地与港澳更紧密经贸关系，加快推进内地对港澳服务贸易自由化进程。落实中央支持香港经济发展、加强内地与香港经济合作的有关政策措施，促进香港经济发展和民生改善。支持澳门建设世界旅游休闲中心，以横琴开发建设为契机，推动澳门经济适度多元发展。推进两岸经济关系制度化进程，加快完成海峡两岸经济合作框架协议后续商谈，进一步提高两岸经贸和产业合作水平。
　　各位代表，做好2014年经济社会发展各项工作，任务艰巨，责任重大。我们要更加紧密地团结在以习近平同志为总书记的党中央周围，高举中国特色社会主义伟大旗帜，以邓小平理论、“三个代表”重要思想、科学发展观为指导，自觉接受全国人大的监督，虚心听取全国政协的意见和建议，坚定信心，振奋精神，开拓创新，攻坚克难，促进经济持续健康发展和社会和谐稳定。
　　附件

　　名词解释

　　1．第四代移动通信。即4G，是伴随移动互联网发展的新一代移动通信技术，可以提供语音业务和高速宽带数据业务。从移动通信发展历程看，2G实现了模拟语音业务向数字语音业务的转变；3G是在语音业务的基础上，增加了移动数据业务；4G是以移动数据业务为主，并向宽带化方向发展，理论峰值传输速率可达1千兆比特／秒。从技术上看，业界一般用长期演进技术 (LTE)指代4G，包括TD-LTE和LTE FDD两种技术。其中，TD-LTE技术由我国大唐电信、中国移动等企业主导制定。
　　2．政府向社会力量购买服务。指通过发挥市场机制作用，把政府直接提供的一部分服务事项，按照一定的方式和程序交由具备条件的社会力量承担，并由政府根据服务数量和质量向其支付费用。购买服务的主体是各级行政机关和参照[公务员法](https://www.pkulaw.com/chl/823ca97b7f55d18cbdfb.html)管理、具有行政管理职能的事业单位。纳入行政编制管理且经费由财政负担的群团组织，也可根据实际需要通过购买服务方式提供公共服务。
　　3．丝绸之路经济带。2013年9月，习近平主席访问中亚四国期间，在哈萨克斯坦纳扎尔巴耶夫大学提出共建丝绸之路经济带的重大倡议。这项倡议旨在传承和发扬团结互信、平等互利、包容互鉴、合作共赢的丝绸之路精神，倡导丝绸之路沿线国家以经济合作为主线，加强政策沟通、道路联通、贸易畅通、货币流通、民心相通，以此推动我国中西部发展和全方位对外开放，促进丝绸之路沿线各国的共同发展与繁荣。
　　4．21世纪海上丝绸之路。海上丝绸之路是古代中国与西方进行贸易、文化交往的海上通道。建设21世纪海上丝绸之路，是2013年10月习近平主席在印度尼西亚国会发表演讲时提出的在新时期、新形势下加强海上对外交往与合作的重大倡议。旨在以“和平、友谊、合作、发展”为核心，加强与周边及沿海国家在互联互通、经贸财金、农业渔业、科技环保、海上安全、社会人文等领域的务实合作，以完善区域联通网、产业带、人文圈，服务国家发展，与有关国家实现和谐相处、心灵相通、互利共赢和共同发展。
　　5．孟中印缅经济走廊和中巴经济走廊。 2013年5月李克强总理访问印度期间，中印两国总理共同倡议建设孟中印缅经济走廊。该走廊以中国昆明为东端，印度加尔各答为西端，包括以缅甸曼德勒、孟加拉国达卡和吉大港等主要城市和港口为关键节点的广大地区。李克强总理访问巴基斯坦期间，双方发表联合声明，明确就中巴经济走廊成立联合工作组，共同研究制订远景规划。该走廊北起中国新疆喀什，南至巴基斯坦瓜达尔港，包括以沿线主要城市和周边地区为关键节点的广大区域。孟中印缅经济走廊和中巴经济走廊建设，拟通过推动相互投资和贸易往来，扩大人文交流，形成促进沿线地区发展的充满活力的经济带。
　　6．纵横联动协同管理机制。是有关部门和地方政府建立的企业投资项目监管体系。发展改革部门负责制定和组织实施发展战略、发展规划、产业政策、总量控制目标。城乡规划、国土资源、环境保护、行业管理等部门负责标准制定和监管。有关部门建立信息互通制度，及时互相抄送核准、审批结果。未取得规划选址、用地预审、环评审批、节能评估审查意见的，不得予以核准；未取得核准及相关审批文件，不予施工许可。加强事中事后监管，大力查处违法违规行为。
　　7．存款保险制度。又称存款保障制度，是金融安全网的重要组成部分，是国家为保护存款人的利益和维护金融稳定，通过立法建立的，在投保机构出现倒闭、破产等规定情形时，由存款保险基金管理机构依法使用存款保险基金向存款人偿付被保险存款、处置问题金融机构的一种制度安排。一般由投保金融机构向存款保险基金管理机构缴纳保险费，存款保险基金管理机构依法采取必要的早期纠正和风险处置措施维护存款保险基金安全。
　　8．存量天然气门站价格。天然气门站是指城市管网(或大用户)接收长输管线天然气的接收站。门站价格为售气、购气双方在门站(即所有权交接点)的交气价格，实行政府指导价，也是最高价，供需双方可在此范围内协商确定具体价格。2013年，按照建立与可替代能源价格挂钩的动态调整机制的思路，将国产陆上天然气、进口管道天然气分为存量气和增量气，出台了天然气价格调整方案。存量气为用户2012年实际使用的气量，增量气为新增加的气量。增量气门站价格按可替代能源(燃料油、液化石油气)加权综合价格85％的水平确定，存量气价格分步调整到位。
　　9．准入前国民待遇加负面清单的管理模式。
　　“准入前国民待遇”是指在投资的整个周期，包括设立、获取、扩大等准入环节，东道国给予外国投资者及其投资的待遇，应不低于给予本国投资者及其投资的待遇。“负面清单”是以列表方式列出针对外资的差别性措施，这些措施不适用于准入前国民待遇，如禁止或限制外资进入的领域等。“准入前国民待遇加负面清单的管理模式”，即除了以负面清单模式列出不符合国民待遇的措施外，在投资的整个周期特别是准入前阶段，东道国给予外国投资者不低于本国投资者的待遇。这种管理方式有利于规范和约束政府行为，为企业创造一个稳定、透明、可预期的营商环境。
　　10．东北和内蒙古大豆、新疆棉花目标价格补贴试点。按照2014年中央一号文件的部署，要积极探索推进农产品价格形成机制与政府补贴脱钩的改革，逐步建立农产品目标价格制度，基本思路是在保护农民利益的前提下，更好地发挥市场在农产品价格形成中的决定性作用。在市场价格过高时补贴低收入消费者，在市场价格低于目标价格时按差价补贴生产者。2014年，先行启动东北和内蒙古大豆、新疆棉花目标价格补贴试点。目标价格按种植成本加基本收益确定，当市场价格低于目标价格时，政府按两者之间的差价对农民进行补贴，确保农民收益稳定。
　　11．农业转移人口市民化成本分担机制。建立健全由政府、企业、个人共同参与的农业转移人口市民化成本分担机制，根据农业转移人口市民化成本分类，明确成本承担主体和支出责任。政府要承担农业转移人口市民化在义务教育、就业服务、基本养老、基本医疗卫生、保障性住房以及市政设施等方面的公共成本。企业要落实农民工与城镇职工同工同酬制度，加大职工技能培训投入，依法为农民工缴纳职工养老、医疗、工伤、失业、生育等社会保险费用。农民工要按照规定承担相关费用，积极参加城镇社会保险、职业教育和技能培训等，提升融入城市社会的能力。
　　12．生态红线管控制度。指通过设定并严守自然资源利用上线、环境质量安全底线和生态功能保障基线，将经济社会发展活动限制在资源环境承载能力之内，倒逼发展方式加快转变和产业转型升级，提高发展的质量和效益。自然资源利用上线是指设定能源(煤炭)、水、土地等资源消耗和环境容量“天花板”，实行总量控制；环境质量安全底线是指将大气、水、土壤等环境质量“只能变好、不能变坏”作为各级政府的环保责任；生态功能保障基线是指为确保重要生态系统主导功能得到有效保护，在重要生态功能区、生态敏感区、脆弱区、禁止开发区等区域划定生态保护空间，做到“数量要增加、质量要提高”。探索建立资源环境承载能力监测预警机制，对资源消耗和环境容量接近承载能力的地区，实行限制性措施。

第十二届全国人民代表大会财政经济委员会关于2013年国民经济和社会发展计划执行情况与2014年国民经济和社会发展计划草案的审查结果报告

十二届全国人大二次会议主席团：
　　第十二届全国人民代表大会第二次会议审查了国务院提出的《关于2013年国民经济和社会发展计划执行情况与2014年国民经济和社会发展计划草案的报告》以及2014年国民经济和社会发展计划草案。全国人民代表大会财政经济委员会在对计划报告和计划草案初步审查的基础上，根据各代表团和有关专门委员会的审查意见，又作了进一步审查。国务院根据审查意见对计划报告作了修改。现将审查结果报告如下。

　　**一、**财政经济委员会认为，2013年，国务院和地方各级人民政府认真落实党中央决策部署和十二届全国人大一次会议提出的各项要求，围绕科学发展主题和转变经济发展方式主线，坚持稳中求进工作总基调，统筹稳增长、调结构、促改革和惠民生，经济运行稳中向好，质量和效益提高，农业生产再获丰收，物价基本稳定，就业完成预期目标，居民收入持续增长，改革开放取得新进展，各项社会事业全面发展，国民经济和社会发展计划执行情况总体是好的。同时也要看到，经济社会发展仍面临不少矛盾和问题，深化改革的任务十分繁重。经济增长的内生动力不足，转方式、调结构力度需进一步加大；部分行业产能严重过剩，一些企业生产经营困难；农业增产、农民增收难度加大；部分地方和行业债务负担较重，金融机构不良资产有所增加；资源消耗和环境污染问题突出，节能减排任务艰巨；部分大城市房价上涨过快。安全生产、收入分配、征地拆迁、食品药品质量等方面还存在不少突出问题。对此，应当高度重视，认真加以解决。

　　**二、**财政经济委员会认为，国务院提出的2014年计划报告与计划草案符合党的十八大、十八届二中、三中全会和中央经济工作会议精神，符合“十二五”规划纲要的要求，符合我国经济社会发展实际，主要目标和工作安排总体可行，建议批准国务院提出的《关于2013年国民经济和社会发展计划执行情况与2014年国民经济和社会发展计划草案的报告》，批准2014年国民经济和社会发展计划草案。

　　**三、**财政经济委员会认为，2014年是落实党的十八届三中全会决定、全面深化改革的第一年，也是实现“十二五”规划目标的重要一年，做好经济社会发展各项工作意义重大。在计划执行过程中，要全面贯彻落实党的十八大、十八届二中、三中全会精神，贯彻落实习近平总书记系列重要讲话精神，按照中央经济工作会议的要求，坚持稳中求进工作总基调，把改革创新贯穿于经济社会发展各个领域各个环节，促进经济持续健康发展和社会和谐稳定。为此，财政经济委员会提出以下建议：
　　（一）全面推进改革开放不断深化，着力激发市场活力。按照中央总体部署和要求，以经济体制改革为重点，努力推动重点领域和关键环节改革取得新突破。加快政府职能转变，充分发挥市场对资源配置的决定性作用。深化国有企业改革，不断完善现代企业制度，积极发展混合所有制经济。进一步放开市场准入，创造良好投资环境，推动民间资本进入金融、石油、电力、铁路、电信、资源开发、公用事业等领域。积极推进资源性产品价格改革。继续推进利率市场化，完善人民币汇率市场化形成机制。积极构建开放型经济新体制，促进国际国内生产要素有序流动和市场深度融合，提高我国在国际经贸规则形成过程中的话语权，加强多边、双边及区域经济合作，积极推进丝绸之路经济带、21世纪海上丝绸之路等对外经济走廊建设，加快培育国际竞争新优势。坚持市场化改革与法制化建设并举，抓紧研究修改相关法律法规，推进依法行政，更好地发挥政府作用。
　　（二）加快发展方式转变，推动经济转型升级。今年稳增长的任务十分艰巨，要坚持扩大内需方针，着力增加有效需求。合理调整国民收入分配格局，努力增加居民收入，大力培育和发展新的消费热点，提高消费对经济增长的拉动作用。保持投资稳定增长，推进结构优化。促进传统产业技术改造和兼并重组，提升产业素质和竞争优势。坚持通过市场竞争实现优胜劣汰，建立有利于落后产能退出的长效机制和市场环境。大力发展战略性新兴产业，促进新兴产业按照市场规律健康发展。加强关键核心技术和共性技术攻关，加快建立主要由市场评价技术创新成果的机制，加强知识产权保护和运用，使创新成果加快转化为现实生产力。完善促进现代服务业发展的各项政策，加快发展研发设计、现代物流、融资租赁、电子商务、节能环保服务、检验检测等重点行业。继续深入实施区域发展总体战略，加快培育新的经济支撑带。完善发展成果考核评价体系，引导各方面把工作重点放在提高经济增长的质量和效益上。
　　（三）夯实农业农村发展基础，促进城乡一体化发展。继续加大“三农”投入，加强农业农村基础设施建设。完善和强化农业支持保护制度，调整和完善农业补贴政策，健全粮食主产区利益补偿机制，多渠道增加农民收入，切实保障粮食安全。支持多种形式适度规模经营，大力培育新型农业经营主体，不断健全农业社会化服务体系。稳定农村土地承包关系，坚持和完善最严格的耕地保护制度，按照依法自愿有偿原则，有序推进承包地经营权流转。推进征地制度改革，缩小征地范围，规范征地程序，加快建立城乡统一的建设用地市场。扎实推进以人为核心的新型城镇化，有序推进农业转移人口市民化，逐步实现城镇基本公共服务覆盖全部常住人口，健全城乡发展一体化体制机制。
　　（四）加强和改善宏观调控，重视防范化解财政金融风险。保持宏观经济政策连续稳定，继续实施好积极的财政政策和稳健的货币政策，完善调控方式和手段，注重与改革措施相衔接，不断增强宏观调控的前瞻性、针对性和协同性。重视防范化解财政金融风险，坚决守住不发生系统性和区域性风险的底线。加强地方政府性债务管理，妥善处理存量债务，规范管理新增债务，建立债务风险预警和化解机制。完善金融监管协调机制，加强对金融机构表外业务风险监管，依法规范理财业务，促进互联网金融健康发展。采取有效措施逐步缓解全社会负债率和金融杠杆率过高问题。提高直接融资比重，鼓励和引导金融机构加大对“三农”、小微企业和结构调整等方面的支持，努力缓解实体经济融资成本高问题。密切监测房地产市场走势，尽快研究建立房地产市场健康发展的长效机制。
　　（五）加大节能减排工作力度，加强生态文明建设。采取更加有效的对策措施，确保“十二五”规划纲要确定的节能减排约束性目标顺利实现。积极调整能源结构，在不断提高能效的同时，尽快落实能源消费总量控制制度，严格控制化石能源消费量过快增长，提高非煤能源和清洁能源所占比重，从源头减少污染物排放。制定实施有针对性的治理措施，着重抓好大气、水体、土壤等环境污染防治，加强对环境污染特别是雾霾成因的科学研究，抓紧解决霾污染问题。创新节能环保工作理念和方式，更多依靠法律手段和税收、价格等经济手段，建立健全节能环保激励约束机制和生态环境损害责任追究制，严格执行生态补偿制度，加大执法和惩处力度。推进节能环保信息公开和透明，强化舆论监督，增强全社会节能环保的积极性和主动性。
　　（六）切实保障和改善民生，维护社会和谐稳定。做好保障和改善民生工作，既要尽力而为又要量力而行，把不断改善民生建立在财力可持续的基础之上。全面推进各级各类教育发展，提高教育资金使用效率，改善贫困地区义务教育薄弱学校基本办学条件，合理均衡配置教育资源。继续实施就业优先战略和更加积极的就业政策，重点抓好高校毕业生就业和化解产能过剩过程中的职工安置工作。尽快完成城乡居民基本医疗保险制度整合，建立统一的城乡居民养老保险制度。继续深化医药卫生、科技、文化、体育等领域改革。落实好调整完善计划生育的政策。妥善应对人口老龄化。做好残疾人和妇女儿童合法权益保障工作。严格食品药品安全监督管理，坚决遏制重特大安全事故发生。继续加强社会建设，不断创新社会治理和公共服务，维护社会和谐稳定。
　　以上报告，请予审议。

全国人民代表大会财政经济委员会
2014年3月9日

© 北大法宝：（[www.pkulaw.com](https://www.pkulaw.com/)) 专业提供法律信息、法学知识和法律软件领域各类解决方案。北大法宝为您提供丰富的参考资料，正式引用法规条文时请与标准文本核对。
欢迎查看所有[产品和服务](https://www.pkulaw.com/helps/2.html)。法宝快讯：[如何快速找到您需要的检索结果？](https://www.pkulaw.com/helps/70.html)[法宝](https://www.pkulaw.com/helps/69.html)V6有何新特色？

扫描二维码阅读原文

**原文链接：**<https://www.pkulaw.com/chl/e6a6f3f78d1b1b1fbdfb.html>
